# Serum Concentration of Selected Angiogenesis-Related Molecules Differs among Molecular Subtypes, Body Mass Index and Menopausal Status in Breast Cancer Patients

**DOI:** 10.3390/jcm11144079

**Published:** 2022-07-14

**Authors:** Dimitrios Balalis, Dimitrios Tsakogiannis, Eleni Kalogera, Stefania Kokkali, Elli Tripodaki, Alexandros Ardavanis, Dimitrios Manatakis, Dionysios Dimas, Nektarios Koufopoulos, Florentia Fostira, Dimitrios Korkolis, Ioannis Misitzis, Nikolaos Vassos, Chara Spiliopoulou, Dimitrios Vlachodimitropoulos, Garyfalia Bletsa, Nikolaos Arkadopoulos

**Affiliations:** 1 Department of Surgical Oncology, Saint-Savvas Anticancer Hospital, 11522 Athens, Greece; dbalalis@gmail.com (D.B.); dkorkolis_2000@yahoo.com (D.K.); 2 Research Center, Hellenic Anticancer Institute, 10680 Athens, Greece; dtsakogiannis@gmail.com (D.T.); kalogerael@yahoo.gr (E.K.); bletsag@yahoo.gr (G.B.); 3 Department of Medicine and Laboratory, Hippokration General Hospital, National and Kapodistrian University of Athens, 11527 Athens, Greece; stefaniakokkali8@gmail.com; 4 Department of Medical Oncology, Saint-Savvas Anticancer Hospital, 11522 Athens, Greece; ellisophia@hotmail.com (E.T.); ardavanis@yahoo.com (A.A.); 5 Department of Surgery, Athens Naval and Veterans Hospital, 11521 Athens, Greece; dmanatak@yahoo.gr; 6 Breast Unit, Psychikon Clinic, Athens Medical Center, 11525 Athens, Greece; dionysis.dimas@gmail.com (D.D.); imissitzis@gmail.com (I.M.); 7 Department of Pathology, Attikon University Hospital, National and Kapodistrian University of Athens, 12462 Athens, Greece; koufonektar@yahoo.com; 8 Molecular Diagnostics Laboratory, Institute of Nuclear & Radiological Sciences and Technology, Energy & Safety (INRASTES), National Center for Scientific Research Demokritos, 15341 Athens, Greece; florentia_fostira@hotmail.com; 9 Medical Faculty Mannheim, University Medical Center Mannheim, University of Heidelberg, 68167 Mannheim, Germany; 10 Department of Forensic Medicine and Toxicology, National and Kapodistrian University of Athens, 11527 Athens, Greece; chspiliop@med.uoa.gr (C.S.); dvlacho@gmail.com (D.V.); 11 Department of Surgery, Attikon University Hospital, National and Kapodistrian University of Athens, 12462 Athens, Greece; narkado@hotmail.com

**Keywords:** angiogenesis, molecules, breast cancer, subtype, body mass index, menopausal status

## Abstract

Background: Angiogenesis is a hallmark of breast cancer (BC) and is mediated by the vascular endothelial growth factor (VEGF) signaling axis. It is regulated by different proangiogenic factors, including platelet-derived growth factor-CC (PDGF-CC) and heparin-binding EGF-like growth factor (HB-EGF), as well as co-receptors, such as neuropilin-1, which could have prognostic implications in BC patients. Patients and methods: We assessed the serum levels of VEGF, HB-EGF, PDGF-CC and neuropilin-1 in 205 patients with early BC (invasive, *n* = 187; in situ, *n* = 18) and in 31 healthy donors (HD) and investigated the potential associations with clinical and histopathological parameters. Results: VEGF serum levels were significantly higher in patients with invasive versus ductal carcinomas in situ. PDGF-CC serum concentrations varied among BC molecular subtypes. Furthermore, we observed a differential expression of most biomarkers between overweight/obese (body mass index (BMI) ≥ 25 kg/m^2^) and non-obese patients among the BC molecular subtypes. Finally, the classification of subjects according to menopausal status revealed a significant difference in specific biomarker levels between patients and HD. Conclusion: The serum concentrations of angiogenic molecules differ among breast cancer molecular subtypes and are affected by the BMI and menopausal status, which could have possible clinical or prognostic implications.

## 1. Introduction

Breast cancer represents the most common cancer in women worldwide and contributed to 25.8% of the total number of new cases diagnosed in 2020. The rate of new cases of female breast cancer is 128.3 per 100,000 women per year [1,2]. Angiogenesis plays a crucial role in both breast tumor growth and distant metastasis [3]. It is mediated by the vascular endothelial growth factor (VEGF) signaling axis and is regulated by different proangiogenic factors, including platelet-derived growth factor (PDGF) and heparin-binding EGF-like growth factor (HB-EGF), as well as co-receptors such as neuropilin-1 (NRP-1) [4].

HB-EGF is a ligand in the epidermal growth factor receptor (EGFR) family [5], and its expression is mostly enhanced in breast cancer tissues compared to other EGFR ligands [6]. Previous studies have shown that HB-EGF plays a crucial role in mammary carcinoma, especially in promoting angiogenesis, local invasion and tumor metastasis [5,7]. Moreover, the elevated expression of HB-EGF is correlated with a higher histological grade, higher rate of lymph node metastasis and worse overall survival in breast cancer patients [6,7]. Furthermore, HB-EGF promotes cancer development in association with proangiogenic platelet-derived growth factors (PDGFs) [5].

The PDGF signaling pathway comprises two kinase receptors, i.e., PDGFRα and PDGFRβ, and five ligands, i.e., PDGF-AA, PDGF-BB, PDGF-AB and PDGF-CC [8,9]. In fact, the overexpression of PDGFs and PDGFRs has been documented in many tumor types, such as gastric, pancreatic, colon, lung, ovarian and breast cancers [9,10]. Specifically, PDGFRα and PDGF-CC have been recently associated with the triple-negative breast cancer (TNBC) subtype, while the PDGF signaling network seems particularly promising for targeted therapies for this breast cancer molecular subtype [8,11]. HB-EGF stimulates the production of VEGF [5,12]. VEGF-A was the first identified proangiogenic factor involved in angiogenesis, lymphangiogenesis and immune response, and at the same time was related with poor prognosis in many cancers, including breast cancer [4,13,14,15]. Furthermore, a correlation between VEGF-C and lymph node metastasis has been established in breast cancer [4]. Several studies have demonstrated an association between VEGF-A and tumor cell proliferation in a mouse model [13], while other studies have indicated the prognostic importance of VEGF expression in the tumor [12].

Lastly, NRP1 and NRP2 belong to the type-1 multifunctional membrane glycoprotein family expressed by cancer cells, whose levels correlate with angiogenesis, invasiveness and poor prognosis [13,15]. In particular, high levels of NRP1 were associated with chemoresistance in breast cancers having negative prognostic correlations [16,17]. Moreover, the expression of NRP2 is related with lymph node metastasis in breast cancer [13,16].

Given the roles of the above-mentioned factors in angiogenesis and breast oncogenesis [3], we assessed herein the serum levels of VEGF, HB-EGF, PDGF and NRP-1 in breast cancer patients. Furthermore, we investigated whether there is an association of these angiogenic factors with each breast cancer molecular subtype, the body mass index (BMI) and menopausal status.

## 2. Patients and Methods

### 2.1. Study Design

The study cohort consisted of 236 females divided into two groups. The first group consisted of 205 female patients with breast cancer (BC) and the second group (the control group) of unaffected women (*n* = 31). The study was conducted in accordance with the principles of the Declaration of Helsinki [18] and was approved by the Ethics Committee of the Cancer Hospital.

### 2.2. Patient Selection and Data

All 205 female patients with breast cancer were recruited from the Breast Unit of St. Savvas Anticancer Hospital of Athens between May 2016 and July 2018. Eligible patients were those who were ≥18 years old, suffered from BC and had undergone primary surgical treatment. Patients with BC having received neoadjuvant therapy, patients with metastatic disease and patients with cancer in areas other than the breast were excluded from this study. On the other side, all women in the control group were recruited during their annual breast examination, which involved clinical examination and mammography.

Clinical and histopathological data were retrieved from the medical records of the BC patients. Breast cancer patients were characterized as having invasive ductal carcinoma (IDC), ductal carcinoma in situ (DCIS) or invasive lobular carcinoma (ILC). A complete histopathology report was obtained for each patient, where estrogen receptors (ERs), progesterone receptors (PRs), human epidermal growth factor receptors (HER2) and the Ki-67 proliferation index were recorded. Based on these data, patients were sub-categorized by molecular subtype (Luminal A, Luminal B, triple-negative or Her2-overexpressing). The BMI was also monitored for both groups. Women with BMI values ≥25 kg/m^2^ and BMI values <25 kg/ m^2^ were classified as overweight/obese and non-obese, respectively.

### 2.3. Sample Preparation

A blood sample was collected from all patients. The blood samples were collected preoperatively and before the patients underwent any kind of neoadjuvant therapy. Peripheral venous blood samples were collected between 08:00 a.m. and 10:00 a.m. into serum separator vacutainers and allowed to clot for 30 min at room temperature. Subsequently, the samples were centrifuged at 3000× *g* for 15 min at 8°C, the serum was isolated, divided into aliquots and stored at −80 °C until being assayed.

### 2.4. Enzyme-Linked Immunosorbent Assay (ELISA)

Serum levels of VEGF, HB-EGF, PDGF-CC and NRP-1 were quantified using an Enzyme-linked immunosorbent assay (Elisa) with the respective Human VEGF, HB-EGF, PDGF-CC and NRP-1 Quantikine^®^ ELISA kits (R&D Systems, Minneapolis, MN, USA) used according to the manufacturer’s instructions. All samples were assayed in duplicate. Finally, the acquired absorbance data were measured using a Multiscan^TM^ FC Microplate Photometer (Thermo Fisher Scientific, Waltham, MA, USA).

### 2.5. Statistical Analysis

Chi-square analysis or the Fisher exact test were used to evaluate the association of VEGF, HB-EGF, PDGF-CC and NRP-1 expression with categorical variables. Normality was examined using the Shapiro–Wilk or Kolmogorov–Smirnov tests. In particular, the Mann–Whitney methodology was used to examine the association of each individual molecule with breast cancer, the different molecular subtypes of breast disease, the BMI and menopausal status, while the Kruskal–Wallis test was performed to compare the medians of multiple groups. All *p* values were regarded as statistically significant at the 0.05 cut-off level. All statistical analyses were carried out using SPSSv25 (SPSS Inc., Chicago, IL, USA) software.

## 3. Results

The mean age of the whole cohort was 58.3 (range 46–71) years and the mean age of the BC group and control group was 59.5 (range 47–71.5) years and 50 (range 41–59) years, respectively. Among the BC patients, 151 (74%) patients were postmenopausal and 54 (26%) were premenopausal. The majority of women in the control group also had a postmenopausal status. The mean BMI value of both the breast cancer cases and the healthy women was 26 ± 3 kg/m^2^. The type of BC was presented as follows: IDC (*n* = 153), DCIS (*n* = 18) and ILC (*n* = 34). Patients were characterized as Luminal A (*n* = 60), Luminal B (Her2-positive) (*n* = 24), Luminal B (Her2-negative) (*n* = 51), triple-negative (*n* = 33) and HER2-positive (ER-negative, PR-negative and HER2-positive) (*n* = 19).

**Figure 1:**
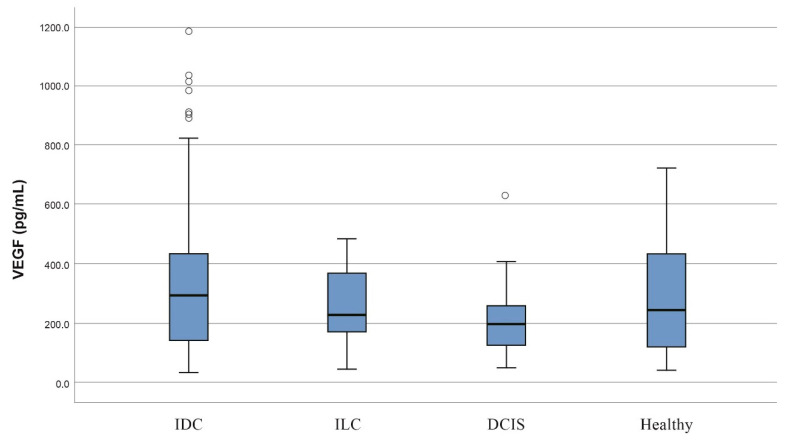
Box plot of serum VEGF levels among patients with different histological types. Serum VEGF levels are significantly higher in invasive ductal carcinoma (IDC) patients compared to ductal carcinoma in situ (DCIS) patients (*p* = 0.022).

Increased serum levels of VEGF and PDGF-CC and decreased levels of HB-EGF and NRP-1 were observed in breast cancer patients when compared to the control group (Table 1). However, these differences did not reach the limits of statistical significance (*p* > 0.05). No significant association concerning the examined protein levels was observed when stratification was made based on the different breast subtypes, i.e., IDC, DCIS and ILC versus the control group. Interestingly, a statistically significant difference in VEGF serum levels was only observed when IDC and DCIS histological types were compared (median concentration; 294.2 versus 197, *p* = 0.022) (Figure 1). The median concentrations of VEGF, HB-EGF, PDGF-CC and NRP-1 are listed in Table 1.

**Figure 2:**
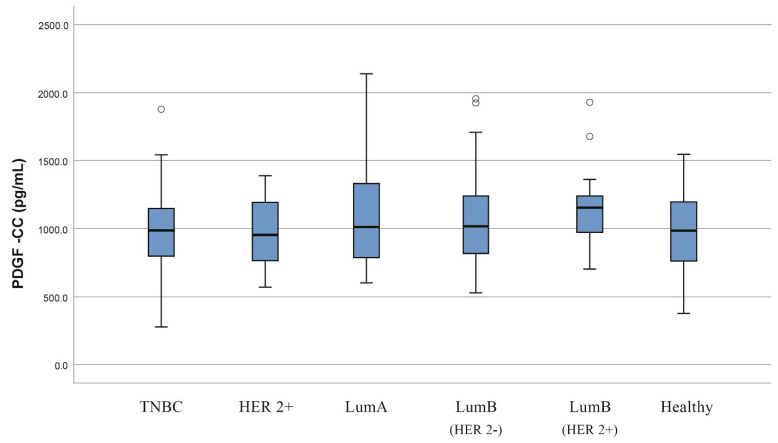
Box plot of serum PDGF-CC levels among patients with different molecular subtypes. Serum PDGF-CC levels are significantly lower in TNBC patients compared to Luminal B (HER2-negative) patients (*p* = 0.031).

Furthermore, our results showed no considerable difference between different molecular subtypes (Luminal A, Luminal B, triple-negative and Her2-positive) and the control group regarding the serum levels of the examined molecules (Table 2). However, a statistically significant higher median serum concentration of PDGF-CC was observed in the Luminal B (Her2-negative) subtype compared to the TNBC molecular subtype (median concentration; 1018 versus 984.7, *p* = 0.031) (Figure 2). Additionally, it has to be mentioned that no statistical association was observed between the tumor grade/stage of disease and the VEGF, HB-EGF, PDGF-CC and NRP-1 serum levels (Table 3).

**Figure 3:**
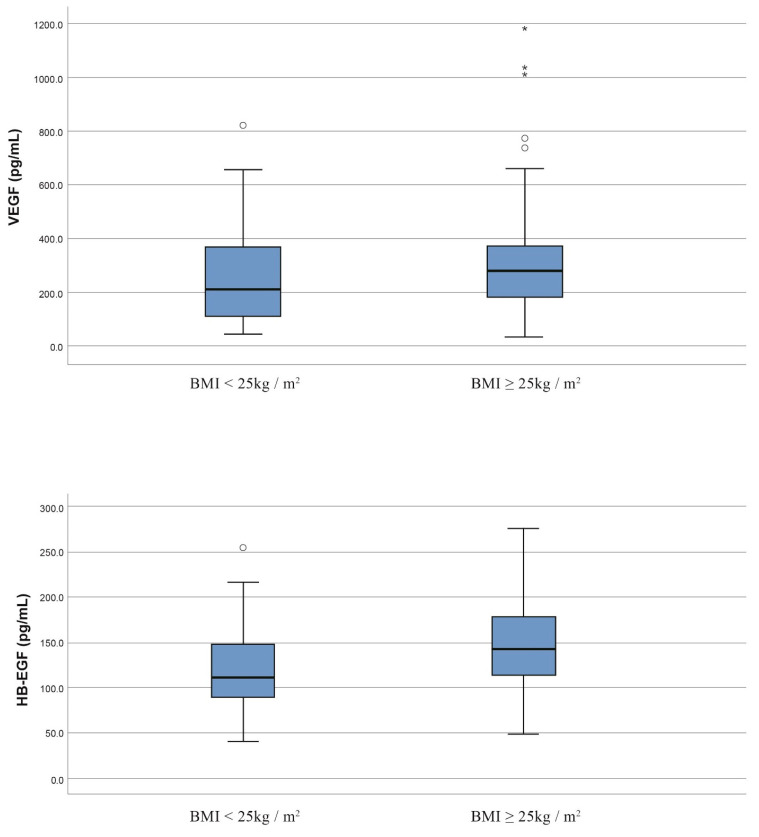
Box plots of VEGF and HB-EGF serum levels according to the BMI status of patients with breast cancer.

The serum levels of VEGF, HB-EGF and PDGF-CC were found to be increased in overweight/obese patients when compared to those of patients with BMI values < 25 kg/m^2^; however, only the differences that were observed in VEGF and HB-EGF serum levels were considered statistically significant (VEGF: median concentration; 280.5 versus 212.2, *p* = 0.049, HB-EGF: median concentration; 142.6 versus 111.3, *p* < 0.001) (Table 4) (Figure 3). Moreover, the concentration of NRP-1 remained relatively invariable between overweight/obese and non-obese breast cancer patients. By merging the outcomes derived from the BMI values and the different breast cancer histological types, it was found that in IDC cases, the VEGF and HB-EGF serum levels were augmented in overweight/obese patients when compared to those of patients with a BMI < 25 kg/ m^2^. However, only the differences in HB-EGF serum levels reached the limits of statistical significance (median concentration; 141.3 versus 116.4, *p* = 0.004). On the other hand, the serum concentrations of PDGF-CC and NRP-1 remained unaffected in IDC cases regarding the BMI values. Considering the ILC group, the serum levels of VEGF, HB-EGF and PDGF-CC were found to be increased in overweight/obese patients; however, the median concentration of VEGF was found to be significantly higher in patients with a BMI ≥ 25 kg/m^2^ compared to that of patients with a BMI < 25 kg/m^2^ (median concentration; 270.2 versus 174.6, *p* = 0.035). On the other hand, the serum levels of NRP-1 were found to be relatively reduced in overweight/obese patients, but these differences did not reach the limits of statistical significance (*p* > 0.05).

**Figure 4:**
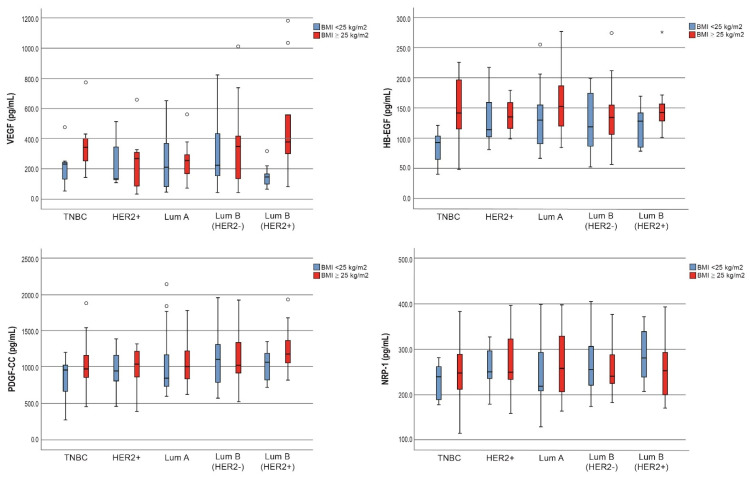
Box plots of VEGF, HB-EGF, PDGF-CC and NRP-1 serum levels among patients with different molecular subtypes according to their BMI status.

Regarding the BMI values among breast cancer molecular subtypes, the Luminal A patients who were overweight/obese had elevated serum levels of VEGF, HB-EGF, PDGF-CC and NRP-1 when compared to the non-obese patients (*p* > 0.05) (Table 4) (Figure 4). This was in accordance with the results among the Luminal B patients, but involved only the VEGF serum levels, i.e., significantly higher in overweight/obese women with a BMI ≥ 25 kg /m^2^ versus a BMI < 25 kg/m^2^ (median concentration; 359.5 versus 193.7, *p* = 0.048). Notably, the aforementioned observations only involved Luminal B (Her2-positive) samples (*p* = 0.008) (Table 4). For TNBC patients, increased serum levels were observed for all the investigated molecules in overweight/obese patients compared to non-obese individuals; however, only differences in HB-EGF serum concentrations were regarded as statistically significant (median concentration; 141.4 versus 92.6, *p* = 0.010) (Table 4).

Regarding the menopausal status of women, the median concentration of HB-EGF was significantly lower in premenopausal breast cancer patients compared to premenopausal healthy women (median concentration; 125.2 versus 144.4, *p* = 0.039). Moreover, the serum levels of PDGF-CC were found to be higher in postmenopausal patients versus postmenopausal women in the control group (median concentration; 984.7 versus 835.8, *p* = 0.04). Nonetheless, after stratification according to menopausal status, the serum levels of VEGF and NRP-1 were not modified between breast cancer patients and the control group (Table 5). Among the histological groups, the HB-EGF serum levels in IDC premenopausal women were significantly lower than those of healthy premenopausal women (median concentration; 130.6 versus 144.4, *p* = 0.023) (Table 6). In addition, the serum levels of PDGF-CC were considerably higher in IDC postmenopausal women than the levels found in the control group (median concentration; 974.0 versus 835.8, *p* = 0.045). However, no significant associations were found concerning the levels of VEGF and NRP-1 between patients diagnosed with IDC and the control group (Table 6). Moreover, no significant molecule concentration changes were observed among IDC, DCIS and ILC, according to the menopausal status of the breast cancer patients. Similarly, no significant associations were demonstrated either between molecular subtypes and the control group, or among the different molecular subtypes concerning the menopausal status of the women and the expression levels of the examined molecules (data not shown).

## 4. Discussion

Angiogenesis represents one of the most important factors in the progression of breast cancer [1,19]. Different growth factors such as VEGFs and PDGFs are responsible for the initiation and progression of tumor angiogenesis in BC [1,3]. The process of angiogenesis depends on the interaction of multiple proteins with proangiogenic properties [20]. The present analysis focuses on the serum protein levels of VEGF, PDGF-CC, HB-EGF and NRP-1 in women diagnosed with breast cancer, in comparison with healthy women. The levels of the proteins were also studied within the various molecular subtypes of breast cancer and according to their BMI and menopausal status.

The absence of statistically significant differences in the concentrations of the examined molecules between breast cancer patients and healthy women is probably due to the modest number of healthy women. However, a statistically significant difference in the concentration of VEGF was found between IDC and DCIS, which supports the idea that the angiogenic pathway plays an important role in tumor progression, as DCIS is considered to be a precursor to IDC.

We demonstrated a discrepancy in PDGF-CC levels between the Luminal B (Her2-negative) and the triple-negative molecular subtypes. More specifically, the median concentration of PDGF-CC was found to be significantly higher in patients with Luminal B (Her2-negative) compared to patients with TNBC. The PDGF-CC ligand was detected towards the end of the 1990s, and it has been proven to be related to tumor growth via paracrine signaling by means of PDGFRa [8,21]. Recent studies have also demonstrated that the increased expression of the PDGFR-CC ligand is correlated with young age, lymphatic metastasis, Her2 expression, a high Ki67 proliferation index, as well as an increased risk for the appearance of distant metastases within a five-year period, which confers poor prognosis for the disease [8,21,22]. In accordance with these findings, we found a higher level of PDGF-CC in the Luminal B (Her2-negative) subgroup, which underlines the different biological and molecular pathways that are involved in each breast cancer subtype. Future studies including a larger number of patients per molecular subtype are required to explore potential associations of the other biomarkers. Furthermore, other studies have shown that the expression of the PDGF-CC ligand is a vital factor for achieving a therapeutic response, since it is highly expressed in tumors resistant to therapies, both anti-VEGF factors and chemotherapy [21,23]. Moreover, recent studies have shown that patients with the triple-negative molecular subtype and low expression of PDGF-CC demonstrate higher survival rates without distant metastases, in comparison to patients that have a high expression of PDGF-CC [8,22].

Overweight and obesity, as measured by a high BMI, increases the risk of postmenopausal breast cancer, with obesity being related to more aggressive tumors, an increased rate of involved lymph nodes and disease recurrences [24,25,26]. According to our results, the levels of VEGF were significantly increased in patients with a BMI ≥ 25 Kg/m^2^ compared to those with a BMI < 25 Kg/m^2^ (*p* = 0.049). In addition, patients with a BMI ≥ 25 Kg/m^2^ demonstrated significantly higher levels of HB-EGF (*p* < 0.001). These results are supported by earlier studies in which the VEGF and HB-EGF serum levels are significantly influenced by an increased BMI [25,27]. Interestingly, the levels of HB-EGF were found to be significantly higher in premenopausal breast cancer patients compared to the healthy donors (*p* = 0.039). HB-EGF is one of the most important proangiogenic factors and serves as a potential therapeutic target for TNBC [21]. However, to our knowledge, an experimental study using mice has explored the extent to which the serum and visceral fat levels of VEGF protein vary according to menopausal status [26]. It was found that VEGF protein levels are increased not only in the serum, but also in the visceral fat in obese postmenopausal mice compared to non-obese postmenopausal mice [26]. Our results demonstrated that the concentration of VEGF protein is not significantly related to menopausal status which is in accordance with the results of this experimental study.

VEGF expression in breast cancer has been correlated with tumor size, a high histological grade, lymph node metastasis, hormone-receptor negativity and Her2 overexpression [28,29]. In a large study of patients suffering from triple-negative breast cancer, an increase in angiogenesis was detected and was closely related to VEGF expression [29]. Moreover, it was demonstrated that VEGF expression was correlated to an even greater extent and with higher frequency in Luminal B (Her2-positive) and triple-negative subtypes, as compared to the Luminal A subtype (*p* < 0.0001) [28,29]. These findings were not confirmed in our analysis.

We detected higher VEGF levels in patients with aggressive triple-negative breast cancer in comparison with other molecular subtypes as well as with the control group. Despite the lack of statistical significance, the presence of higher VEGF levels in this molecular subtype is of great interest since new VEGF inhibitors have recently been considered to be effective therapeutic options [30]. More specifically, VEGF levels were retrospectively assessed in 679 breast cancer patients, and it was shown that VEGF levels in the triple-negative molecular subtype (*n* = 87) were significantly increased compared to patients with other molecular subtypes (*p* < 0.0001) [31].

Among the patients in the triple-negative molecular subgroup, we detected no significant difference between overweight/obese and normal-weight patients. On the contrary, among the patients with the Luminal B molecular subtype, the VEGF serum concentration was increased in overweight/obese patients compared to women with a BMI < 25 kg/m^2^. Specifically, the relationship between obesity and VEGFs showed statistical significance in patients having Luminal B (Her2-positive) molecular subtype. Similarly, we demonstrated that HB-EGF levels were increased in overweight/obese patients as opposed to regular-weight patients within the triple-negative molecular subtype group, and the difference was statistically significant (*p* = 0.010). Further investigations are needed to explain this difference since HB-EGF inhibition leads to decreased tumor growth in TNBC [5].

There are some limitations arising in the current study. Apart from the relatively limited number of patients per breast cancer subtype, we reported the serum levels of proteins involved in angiogenesis, whereas data on the tissue levels of these molecules are lacking. Therefore, we intend to evaluate the expression of VEGF, HB-EGF, PDGF-CC and NRP-1 in formalin-fixed paraffin-embedded (FFPE) tissue specimens. Besides this, we did not report any potential correlations between the serum levels in the different molecules and the prognostic parameters due to the short follow-up.

## 5. Conclusions

Our results support our initial hypothesis that growth factors involved in angiogenesis are associated with the molecular subtypes of breast cancer. Some of these growth factors are also associated with menopausal status and the BMI of breast cancer patients. Additionally, the present study suggests a possible impact of obesity on the adverse effects of HB-EGF on breast cancer in general and in the triple-negative molecular subtype. The mentioned angiogenesis-related molecules could also be used as predictive markers for breast cancer patients, but further studies are needed.

## Figures and Tables

**Table 1:** Median concentrations (interquartile range) of VEGF, HB-EGF, PDGF-CC and NRP-1 in the serum of the examined patients and healthy women.

	Healthy	Breast Cancer Patients	
	Median (Interquartile Range)	Median (Interquartile Range)	*p*-Value
Total	N = 31	N = 205	
VEGF (pg/mL)	242.8 (113–437.4)	270.8 (144.3–407)	0.652
HB-EGF (pg/mL)	142.3 (118.8–173.7)	128.9 (100.5–172.6)	0.152
PDGF-CC (pg/mL)	985.8 (752.9–1203)	1032.5 (824–1222.5)	0.333
NRP-1 (pg/mL)	264.9 (194.2–311.5)	257.7 (218.3–301.1)	0.698

**Table 2:** Median concentrations of VEGF, HB-EGF, PDGF-CC and NRP-1 in the serum of the examined patients considering breast cancer molecular subtypes.

		VEGF (pg/mL)	HB-EGF (pg/mL)	PDGF-CC (pg/mL)	NEUROPILIN-1 (pg/mL)
	Ν	Median (Interquartile Range)	Median (Interquartile Range)	Median (Interquartile Range)	Median (Interquartile Range)
Total					
Luminal A	60	249.3 (159.9–377.7)	137.7 (102.8–169.9)	1004 (776.7–1350.3)	261.1 (214.1–312.3)
Luminal B	75	316.5 (145.4–449)	128.4 (99–158.6)	1060 (836.9–1263)	255 (221.2–305.8)
Luminal B (HER2−)	51	337.2 (166.4–449)	121.2 (98.9–157.8)	1018 (793.3–1263)	245.1 (221.2–294)
Luminal B (HER2+)	24	261.6 (133.5–521.9)	138.2 (102.4–166.6)	1156 (968.1–1254.5)	274.3 (225.1–333.4)
Triple Negative	33	337.9 (198.9–478.8)	121.8 (89.3–189.4)	984.7 (768.8–1161.5)	242.3 (199.2–288.5)
HER2+	19	273.6 (114.3–380.7)	117.9 (102.4–158.8)	951.7 (736.7–1220)	250.1 (231.7–323)
Healthy	31	242.8 (113–437.4)	142.3 (118.8–173.7)	985.8 (752.9–1203)	264.9 (194.2–311.5)

**Table 3:** Correlation of VEGF, HB-EGF, PDGF-CC and neuropilin-1 expression levels with tumor grade and stage of disease.

	**VEGF**		**HB-EGF**	
	**Median** **(Interquartile Range)**	***p*-Value**	**Median** **(Interquartile Range)**	***p*-Value**
**Grade**		0.757		0.551
1	303.0 (114.4–437.7)		125.5 (87.1–152.3)	
2	266.2 (145.4–398.8)		129.3 (101.1–165.4)	
3	311.5 (143.5–416.4)		127.7 (99.4–178.2)	
**Stage**		0.365		0.219
Ι	310 (166–431)		129 (87–160)	
ΙΙ	229 (100–374)		118 (101–158)	
ΙΙΙ	337 (216–398)		142 (124–210)	
ΙV	267 (130–472)		119 (91–172)	
	**PDGF-CC**		**NEUROPILIN-1**	
	**Median** **(Interquartile Range)**	***p*-Value**	**Median** **(Interquartile Range)**	***p*-Value**
**Grade**		0.731		0.585
1	948.7 (719.8–1265.0)		271.3 (249.3–284.3)	
2	1001.0 (793.3–1215.0)		247.3 (213.7–300.5)	
3	1041.0 (850.8–1201.0)		264.1 (223.8–321.9)	
**Stage**		0.169		0.947
Ι	965 (762–1.189)		264 (215–295)	
ΙΙ	1.009 (905–1.188)		260 (234–290)	
ΙΙΙ	1.171 (934–1.354)		250 (206–322)	
ΙV	1.007 (720–1.143)		242 (218–350)	

**Table 4:** Differences in molecule levels considering BMI values and histological types/molecular subtypes of breast cancer.

	BMI		
	BMI < 25 kg/m^2^	BMI ≥ 25 kg/m^2^	
	Median (Interquartile Range)	Median (Interquartile Range)	*p*-Value
***Healthy***			
**VEGF**	211.1 (91.6–315.3)	292.9 (156.3–502.1)	0.224
**HB-EGF**	130.4 (108.8–151.1)	155.2 (125.9–190.0)	0.077
**PDGF-CC**	1026.5 (631.3–1240.8)	938.9 (759.3–1126.0)	0.790
**NEUROPILIN-1**	303.8 (193.3–332.7)	233.8 (190.4–270.2)	0.142
***Breast Cancer***			
**VEGF**	212.2 (104.2–375.2)	280.5 (168.7–374.2)	0.049
**HB-EGF**	111.3 (88.1–149.2)	142.6 (112.2–179.8)	<0.001
**PDGF-CC**	1009.0 (758.1–1211.0)	1034.0 (885.0–1315.0)	0.275
**NEUROPILIN-1**	254.5 (214.4–313.6)	249.8 (213.7–300.5)	0.670
***IDC***			
**VEGF**	212.9 (123.3–385.7)	306.7 (185.3–402.5)	0.084
**HB-EGF**	116.4 (87.4–152.2)	141.3 (109.2–181.5)	0.004
**PDGF-CC**	1018.5 (742.8–1206.0)	1027.5 (874.0–1244.0)	0.493
**NEUROPILIN-1**	251.5 (214.4–303.1)	248.3 (220.5–301.8)	0.908
***ILC***			
**VEGF**	174.6 (72.3–271.8)	270.2 (227.8–371.4)	0.035
**HB-EGF**	110.2 (95.7–173.6)	129.3 (113.7–162.2)	0.376
**PDGF-CC**	985.4 (739.5–1267.8)	1034.0 (867.7–1560.5)	0.376
**NEUROPILIN-1**	238.7 (213.8–321.9)	213.7 (185.3–321.8)	0.295
***Lum B (HER2+)***			
**VEGF**	145.4 (91.2–193.9)	379.4 (256.9–795.5)	0.008
**HB-EGF**	128.2 (92.6–144.7)	142.6 (117.4–163.8)	0.094
**PDGF-CC**	1065.0 (808.4–1229.0)	1183.0 (988.4–1522.5)	0.161
**NEUROPILIN-1**	279.6 (229.4–351.9)	252.5 (185.4–305.5)	0.297
***Lum B (HER2−)***			
**VEGF**	224.4 (128.4–471.5)	348.4 (126.1–424.8)	0.626
**HB-EGF**	118.7 (86.4–174.2)	134.4 (105.3–159.5)	0.516
**PDGF-CC**	1108.0 (790.5–1315.0)	1025.0 (905.9–1346.8)	0.850
**NEUROPILIN-1**	255.0 (214.6–329.6)	240.5 (223.2–288.8)	0.588
***HER2+***			
**VEGF**	135.8 (128.0–380.7)	268.9 (74.4–319.4)	0.536
**HB-EGF**	114.0 (102.4–189.6)	134.8 (110.7–161.7)	0.536
**PDGF-CC**	951.7 (685.5–1171.0)	1041.0 (799.6–1256.0)	0.999
**NEUROPILIN-1**	250.1 (230.5–323.0)	249.8 (232.7–332.2)	0.837
***TN***			
**VEGF**	232.4 (82.7–252.1)	343.7 (240.6–412.9)	0.083
**HB-EGF**	92.6 (56.0–111.1)	141.4 (110.1–198.9)	0.010
**PDGF-CC**	960.8 (596.0–1043.0)	974.3 (827.4–1164.8)	0.182
**NEUROPILIN-1**	238.6 (183.2–267.9)	247.1 (205.3–290.8)	0.299

**Table 5:** The median concentrations (interquartile range) of the examined molecules in premenopausal and postmenopausal women.

	Healthy	Breast Cancer Patients	
	Median (Interquartile Range)	Median (Interquartile Range)	*p*-Value
Premenopause			
VEGF (pg/mL)	239.2 (123.3–413.4)	240 (128.4–317.6)	0.988
HB-EGF (pg/mL)	144.4 (137.1–176.5)	125.2 (94.7–171.2)	0.039
PDGF-CC (pg/mL)	1048 (920.3–1228)	1077.5 (885–1265)	0.981
NRP-1 (pg/mL)	271.1 (207.1–324.1)	254.8 (210.6–293.4)	0.278
Postmenopause			
VEGF (pg/mL)	259.9 (98–523.6)	287.5 (161.8–409.2)	0.906
HB-EGF (pg/mL)	120.7 (114–162.1)	129.3 (102.4–174.2)	0.995
PDGF-CC (pg/mL)	835.8 (622.6–1105)	984.7 (800.6–1199)	0.040
NRP-1 (pg/mL)	237.7 (189.1–291.7)	260.4 (221.2–305.8)	0.129

**Table 6:** *p* values were determined in order to investigate the differences in protein levels between breast cancer histological types and control group as well as among the different histological types when considering the menopausal status of the examined women.

	VEGF	HB-EGF	PDGF-CC	NRP-1
Premenopause	*p*-Value	*p*-Value	*p*-Value	*p*-Value
IDC vs. Healthy	0.674	0.023	0.769	0.355
ILC vs. Healthy	0.634	0.396	0.711	0.220
DCIS vs. Healthy	0.493	0.543	0.543	0.880
IDC vs. ILC	0.485	0.566	0.816	0.545
IDC vs. DCIS	0.328	0.682	0.350	0.620
ILC vs. DCIS	0.438	0.999	0.898	0.438
**Postmenopause**				
IDC vs. Healthy	0.690	0.956	0.045	0.144
ILC vs. Healthy	0.575	0.360	0.227	0.227
DCIS vs. Healthy	0.462	0.432	0.076	0.145
IDC vs. ILC	0.136	0.202	0.390	0.830
IDC vs. DCIS	0.107	0.319	0.619	0.340
ILC vs. DCIS	0.657	0.094	0.363	0.511

## Data Availability

The data presented in this study are available upon reasonable request from the corresponding author.
